# Comparison of different treatment options in submacular haemorrhage

**DOI:** 10.1186/s12886-024-03794-y

**Published:** 2024-12-09

**Authors:** Anna Hillenmayer, Christian M. Wertheimer, Marlene Hillenmayer, Laura D. Strehle, Lennart M. Hartmann, Efstathios Vounotrypidis, Armin Wolf

**Affiliations:** Department of Ophthalmology, University of Ulm, Prittwitzstr. 43, 89075 Ulm, Germany

**Keywords:** Submacular haemorrhage, RtPA, Macular degeneration, Macular haemorrhage, Pneumatic displacement, Subretinal haemorrhage, Pars plana vitrectomy, Subretinal lavage, Recombinant Plasminogen activator

## Abstract

**Background/aims:**

Submacular haemorrhages (SMH) cause significant visual impairment. Until now, the comparative effectiveness of different treatment approaches remains inconclusive without clear treatment guidelines. The aim of our study was to evaluate the effectiveness of 5 surgical treatment modalities in terms of visual prognosis and success rate.

**Methods:**

This retrospective study included 201 patients with SMH. Primary endpoint was best corrected visual acuity (BCVA), secondary endpoints included haemorrhage size and complications. Group 1 was treated with pneumatic displacement and rtPA-injection. Group 2 followed the "Manchester protocol" with rtPA-injection and—if needed—a standardised secondary procedure with pars plana vitrectomy (ppV) and subretinal rtPA. Group 3 underwent vitrectomy with subretinal rtPA, group 4 vitrectomy only and group 5 received subretinal lavage.

**Results:**

Baseline characteristics were a mean age of 79 years and a follow-up of 4.6 months. Pre-intervention BCVA of 1.7 logMAR improved to 1.4 logMAR at follow-up. A gain of > 0.2 logMAR was achieved in 47% of patients, while 20% lost > 0.2 logMAR. Only group 2 achieved a statistically significant visual gain. While group 5 was statistically larger in haemorrhage size preoperatively (*p* < 0.05), all groups were statistically equal in SMH size at follow-up. Complications led to additional interventions in 20% of patients.

**Conclusions:**

No significant change in visual prognosis could be achieved depending on the intervention. As more invasive techniques seem to lack the benefit of a better postoperative prognosis while carrying higher risks, it may be beneficial considering a less invasive option first.

**Supplementary Information:**

The online version contains supplementary material available at 10.1186/s12886-024-03794-y.

## Introduction

Submacular haemorrhage (SMH) is a serious complication of various ocular pathologies, including age-related macular degeneration, trauma, myopic degeneration and retinal vascular disease [1]. The accumulation of blood in the subretinal and sub-RPE layers disrupts the normal function of the macula leading to cell death of the inner retinal layers with a rapid decline in visual acuity and often irreversible damage [1]. Following the haemorrhage, the breakdown of subretinal erythrocytes releases toxic iron [2] and fibrin and extracellular matrix contraction cause scar formation [3]. In animal models, toxicity of blood via iron occurs after short term exposure. Therefore, most authors favor a timely and effective treatment [4]. Until now, subretinal haemorrhage is associated with poor visual and anatomical recovery [5].

To prevent this irreversible visual impairment, several therapeutic approaches have been explored over the years [6]. The primary goal of most treatment modalities is to remove the blood clot from the submacular space and protect the retinal pigment epithelium and photoreceptor cells from the toxic effects of iron [1]. Current treatment options for smaller hemorrhages include observation and anti-VEGF injections [5, 7, 8], whereas for SMH larger than 3–4 papillary diameters, the most preferred treatment options include pneumatic displacement [9], intravitreal injection of tissue plasminogen activator (rtPA) [10, 11], surgical drainage and vitrectomy with or without subretinal rtPA injection [12–14]. However, each approach has its limitations and risks, as well as varying invasiveness and visual prognosis [5, 6]. Most often, the choice of treatment modality depends on several subjective factors such as the size of the haemorrhage, underlying pathology, duration of onset and individual treatment preferences, as well as patient-related factors such as compliance and comorbidities [5, 6, 15]. Due to the heterogeneity of treatment protocols and developing treatment techniques, no clear treatment guidelines have been established to date, and while smaller retrospective analyses exist, randomized controlled trials have not been published in larger numbers or looked at only selected treatment options [6, 10, 16–18].

To better understand the efficacy and safety profiles of several treatment modalities, an analysis comparing their outcomes and their risk profile seems necessary to aid in treatment selection [6]. To assist in the establishment of a potential treatment guideline in our setting, we retrospectively analyzed five different treatment modalities performed on 201 patients diagnosed with SMH at the first presentation performed from 2010–2022. Anatomical and visual outcomes were evaluated and compared in terms of their efficacy, invasiveness and complication rates.

## Methods

Patients with submacular, center involving haemorrhage treated at the Department of Ophthalmology, University Hospital of Ulm, Germany, between 2010 and 2022 were retrospectively analyzed. The study was approved by the Institutional Ethics Committee (460/22) and adhered to the Declaration of Helsinki.

The inclusion criteria targeted patients who experienced functional deterioration due to submacular hemorrhage involving the fovea, specifically those presenting at our center within 14 days of subjective symptom onset. This timeframe was chosen to guarantee a potential for improvement before the onset of fibrosis. Patients were eligible for analysis if they underwent an intervention for subretinal hemorrhage, which included vitrectomy with or without subretinal or intravitreal injection of rtPA and gas fill or subretinal lavage, or intravitreal rtPA combined with expansile gas fill.

The exclusion criteria encompassed patients who were treated solely with anti-VEGF therapy, those who received no treatment, and individuals with incomplete documented data.

**Fig. 1:**
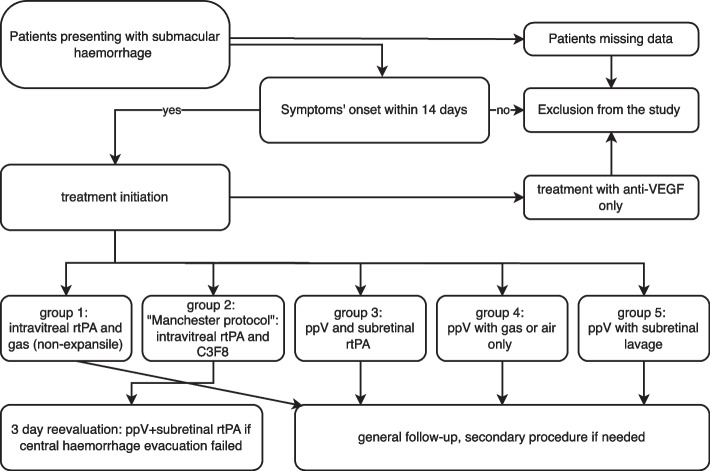
Patients presenting with submacular haemorrhage within 14 days of symptoms onset and treatment initiation other than anti-VEGF only were defined within five subgroups for comparison. Patients with missing data or decision of no treatment were excluded from the study

For analysis and comparison, five subgroups were established according to the type of intervention previously chosen by the treating physician. Individuals in group 1 were treated with intravitreal injections of recombinant tissue plasminogen activator (rtPA) and gas at non-expansile or minimal expansile (SF6 at 20%) concentrations. Group 2 was treated according to a strict treatment protocol (“Manchester Protocol”), where the initial treatment was an intravitreal injection of rtPA and C3F8 gas within 14 days of symptom onset and strict head-down positioning for three days. If the haemorrhage remained within the foveal area after three days of intervention, pars plana vitrectomy (PPV) with subretinal injection of rtPA and C3F8 gas was performed following a strict protocol-detailed evaluation three days later. Group 3 consisted of data from patients treated with ppV and gas with subretinal rtPA as first-line therapy. Group 4 received ppV with gas or air without rtPA and group 5 included patients treated with ppV with or without subretinal rtPA and subretinal lavage of the haemorrhage as primary intervention. The analysis was performed for all included patients as well as the subgroups for comparison (Fig. 1).

For all cases evaluated, the baseline for data documentation was day of initial presentation. The diagnosis of SMH with or without sub-RPE haemorrhage was made clinically and by means of optical coherence tomography (OCT) at initial presentation if available and possible at time of presentation. The location, extent, and exact anatomic site of the haemorrhage were documented by OCT scans and color fundus photographs and at the discretion of the investigating physician. However, preoperative imaging was inconsistent due to image quality limitations resulting from the nature of the underlying pathology. Demographics (age, sex) as well as best corrected visual acuity (BCVA) in Snellen, SMH expansion pre- and post-interventional, complications, and need for further treatment were exported from the medical record files.

The primary endpoint was the final documented BCVA at last follow-up. Snellen BCVA was converted to logarithm of the minimum angle of resolution (logMAR) scale for statistical purposes. Finger counting was converted to 1.9, hand movements to 2.3, and light perception to 2.7 [19]. Visual improvement was defined as significant if BCVA improved by 0,2 logMAR during follow-up compared to baseline pre-interventional BCVA. Secondary endpoints were complication rate, need for secondary interventions, size and extent of submacular haemorrhage as measured by papillary disc size assessed by the investigating physician or by OCT and fundus images if available and evaluated by a blinded observer retrospectively. OCT scans were obtained with the Spectralis HRA + OCT (Heidelberg Engineering GmbH, Heidelberg, Germany) or Cirrus HD-OCT 5000 (Zeiss, Oberkochen, Germany) and fundus photographs with the CLARUS 500 (Zeiss).

### Statistical analysis

Excel 365 (Microsoft, Redmond, WA, USA) and SPSS 29 (IBM, Armonk, New York, USA) were used for data processing and statistical analysis. All errors are expressed as standard deviation. After testing and confirming normal distribution with the Kolmogorov–Smirnov test, statistical comparisons between two groups were made using the t-test, and between more than two groups using ANOVA with Tukey's post hoc test. Pearson's chi-squared cross-tabulation test was used to determine statistical differences in qualitative categorical data. A *p*-value < 0.05 was considered statistically significant. Graphs were generated using GraphPad Prism 10 (GraphPad Software, San Diego, CA, USA).

## Results

### Baseline characteristics

Two-hundred and one eyes with submacular haemorrhage were included in this retrospective study and received one of five interventions. Mean follow-up was 4 (± 6) months. The patients’ mean age was 79 (± 10) years and 113 (56%) were biologically female. The underlying disease was neovascular age-related macular degeneration (AMD) in 147 (73%), retinal macroaneurysm in 19 (9%), peripheral exudative haemorrhagic chorioretinopathy in 8 (4%) and no apparent reason or suspected Valsalva retinopathy in 27 (13%) of the patients. Due to the lack of a standard protocol for treating patients, 28 (14%) received rtPA without a standard protocol, 45 (22%) received the “Manchester protocol”, 73 (36%) received ppV and subretinal rtpA, 43 (21%) received ppV only and 12 (6%) received ppV and subretinal lavage (Table 1).

**Table 1:** Baseline characteristics of patients with submacular hemorrhage prior to treatment. The table summarizes preoperative data across five treatment groups. AMD was the most prevalent condition, with 73% of patients affected. The average age was 79 years, and 56% of the cohort were female. Mean preoperative BCVA (logMAR) was worst in the ppV only group (2.1). The subretinal lavage group had the largest mean hemorrhage size (74 optic disc diameters) Vitreous hemorrhage was most frequent in the ppV only group (65%)

**Preoperative Baseline characteristics**	**All**	**rtPA + Gas**	**Manchester**	**ppV + rtpa**	**ppV only**	s**ubretinal** **Lavage**
**Number of Patients**	201	28	45	73	43	12
**Age**	79	80	78	81	78	78
**Sex (Female)**	113	15	23	48	21	5
**Disease entity: AMD**	147	24	36	51	26	9
**Aneurysm**	19	0	4	10	5	0
PECHR	8	1	1	2	4	0
**other**	27	2	4	9	9	3
**BCVA (logMAR)**	1.7	1.7	1.6	1.6	2.1	1.9
**BCVA (logMAR) AMD**	2.0	1.7	1.6	1.6	2.3	1.9
**SMH size (optic disc diameter)**	28	10	14	21	20	74
**SMH size AMD**	30	10	15	23	29	74
**SMH thickness (µm)**	477	409	477	454	552	424
**Vitreous haemorrhage (%)**	38	4	7	10	65	8

### Visual acuity

**Fig. 2:**
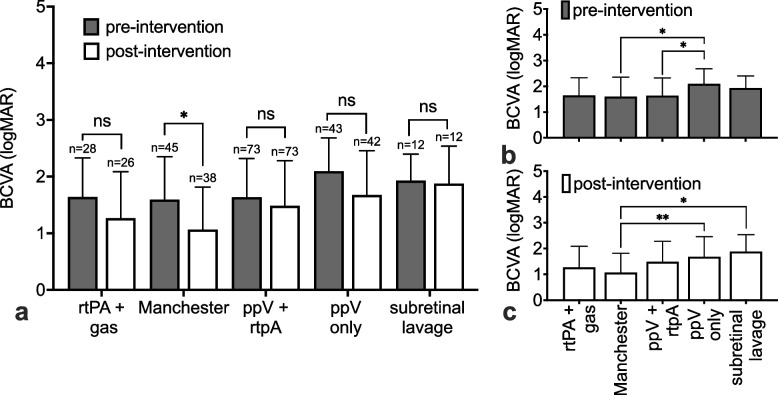
The mean absolute visual acuity was compared between the groups. Only those treated with the Manchester protocol achieved a statistically significant difference, improving from 1.6 (± 0.8) to 1.1 (± 0.8) logMAR (*p* = 0.04). **a** The resulting absolute BCVA was also higher within the Manchester protocol compared to most of the other groups. **c** Pre-interventional BCVA was lower in the ppV only group because of a suspected bias due to a significant higher amount of vitreous haemorrhage in this group (*p < 0.05, **p < 0.01) (**b**)

**Fig. 3:**
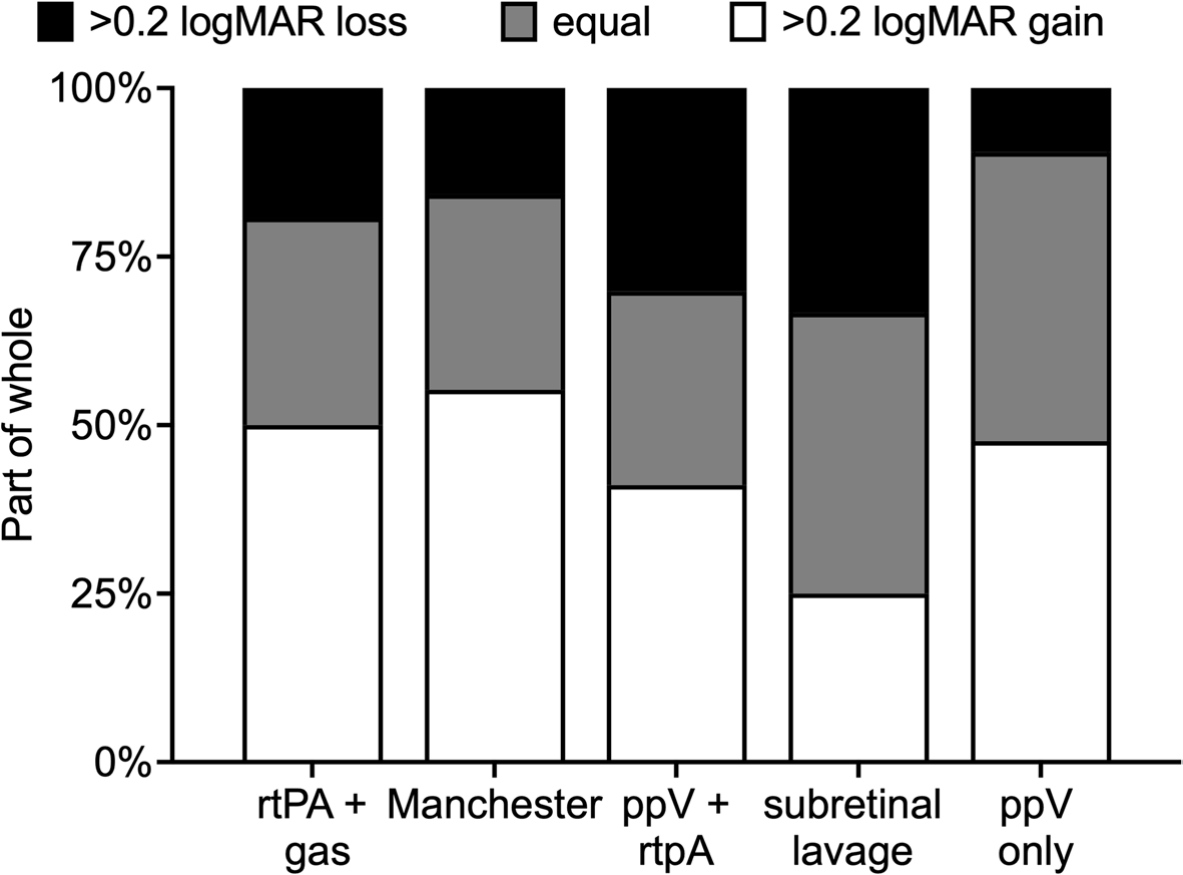
46% of the patients across all groups gained > 0.2 logMAR of visual acuity. However, most patients remained unchanged in BCVA (33%) or lost > 0.2 logMAR of visual acuity at follow-up (21%). The proportions varied between the treatment protocols, but did not reach statistical significance (*p* = 0.2)

Overall, mean BCVA of all treated patients improved statistically significantly from 1.7 (± 0.7) logMAR at baseline to 1.4 (± 0.8) logMAR at follow-up (*p* < 0.001). However, looking at each group individually, only those treated with the Manchester protocol achieved a statistically significant difference, improving from 1.6 (± 0.8) to 1.1 (± 0.8) logMAR (*p* = 0.04). The final BCVA was also better with the Manchester protocol compared to ppV alone (*p* < 0.01) and ppV and subretinal lavage (*p* < 0.05). Of note, baseline BCVA was statistically significantly worse in the ppV only group compared to the other groups (*p* < 0.05), which may be due to a statistically significantly higher proportion of vitreous haemorrhages in this group (65%) compared to all other patients (8%; *p* < 0.001; Fig. 2). Although the gain and resulting absolute BCVA is poor when comparing mean absolute values, there is a proportion of 46% of patients across all groups who gained > 0.2 logMAR of visual acuity. However, 33% of the patients remained unchanged in BCVA and 21% lost > 0.2 logMAR of visual acuity at follow-up. The proportions varied between the treatment protocols, but did not reach any statistical significance (*p* = 0.2) (Fig. 3).

### Haemorrhage size

Due to the inability of central fixation, the retrospective nature of the study and postoperative intravitreal gas tamponade, OCT scans were preoperatively conducted in 47% percent and postinterventional in 30% of the cases, yet in those obtained the central retinal thickness decreased from 477 (± 201) µm to 300 (± 182) µm (*p* < 0.001). Fundus photography was obtained in 127 cases (64%) preoperatively and in 110 cases (55%) for the last follow up. (Supplementary Fig. 1).

**Fig. 4:**
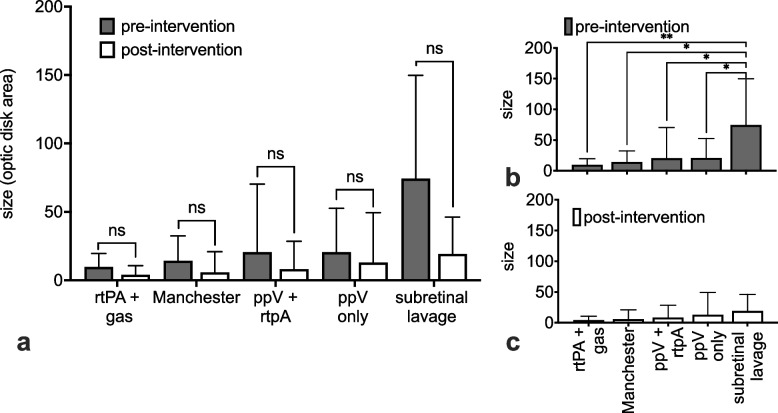
Projected size of haemorrhage measured as optic disc area equivalents on funduscopic images. None of the groups reached statistical significance in the reduction of haemorrhgae size (**a**). Preoperatively, the haemorrhage size was statistically significantly different between groups. **b** However, at final follow-up, the size of haemorrhage was statistically equal between all groups (*p* > 0.05). (*p < 0.05, **p < 0.01) (**c**)

The pre-intervention size of submacular haemorrhages, as measured by optic disc area projection, was significantly larger in the ppV and subretinal lavage group compared to all other groups (*p* = 0.006 to 0.05, depending on the comparison). However, none of the groups reached statistical significance in the reduction of haemorrhage size, most likely due to the large variation in its preoperative size and at the final follow-up, the size of haemorrhage was statistically equal between groups (*p* > 0.05) (Fig. 4).

### Complications

All treatments had high rates of follow- up intervention and complications after the initial procedure. In addition to the 14 (7%) of patients with high intraocular pressure, 40 (20%) underwent repeated ppV due to complications such as 12 postoperative vitreous haemorrhages (6%) and 2 retinal detachments (1%). Most of the other patients had persistent submacular haemorrhages and ppV was attempted as a rescue therapy. Anti-VEGF continuation or therapy initiation was advised in most patients, but was not started within a four-week postoperative time period and followed a treat-and-extend regimen from there on out if BCVA was above 1.3 logMAR. 20 (10%) underwent cataract surgery during the follow-up period (Table 2).

**Table 2:** Comparative outcomes of the five treatment regimens at last follow-up (average 4 months). BCVA (logMAR) and SMH size/thickness varied across groups, with the Manchester Protocol group showing the best visual outcomes for AMD patients. The rtPA + Gas group had the lowest complication and reoperation rates, while subretinal lavage had the largest hemorrhage size and highest complication rates

**Characteristics at last follow-up (4 month in average)**	All	rtPA + Gas	Manchester	ppV + rtpa	ppV only	subretinal Lavage
**BCVA (logMAR)**	1.4	1.1	1.5	1.7	1.7	1.9
**BCVA (logMAR) AMD**	1.5	1.2	1.1	1.5	2.0	1.9
**SMH size (optic disc diameter)**	8	4	6	5	11	12
**SMH size AMD**	11	4	6	7	18	19
**SMH thickness (µm)**	262	222	285	251	423	129
**Re-ppV (%)**	20	15	31	16	14	33
**Cataract surgery** (%)	10	4	11	11	9	17
**Complications (%)**	14	7	9	22	9	25

## Discussion

Until now, subfoveal haemorrhages remain a sight-threatening condition. The management is not standardized and various treatment modalities are described in the literature [5, 13, 17, 20]. Mostly, factors such as size, thickness, duration and location are considered for choice of treatment, yet the choice also depends on the clinical situation, patient-related factors and the preferences of the treating ophthalmologist [21, 22]. At present, the therapeutic options vary, but all with limited success. Studies evaluating their success-to-risk ratio are therefore necessary to help balance the risk of intervention and invasiveness with the prognosis of visual improvement [6]. With 201 eyes included, this is one of the largest monocentric retrospective comparative effectiveness studies of five most established invasive treatment modalities for SMH [5, 6].

Observed treatment outcomes in this study was a slight improvement in BCVA in all treatment groups, regardless of the modality used with 46% of patients in all groups gaining more than 0.2 logMAR. Divided by groups however, only those treated with the Manchester protocol showed a statistically significant improvement. Interestingly, the mean lower preinterventional BCVA in some groups did not lead to a statistically significant worse postinterventional BCVA during follow-up compared to the other groups. Our results regarding visual recovery are consistent with other studies [10, 23]. Boral et al. showed that one third of 43 patients experienced a gain of two or more lines and in a group of 103 patients who underwent more invasive treatment options improved from 2.06 to 0.96 logMAR [24]. However, in most studies, vision remained poor after surgery [10]. In a meta-analysis of 12 different trials, final visual acuity ranged from 0.42 to 1.73 logMAR, despite improvement between pre- and post-operative scores [23]. Although the mean visual acuity improved after treatment in our study, the overall visual prognosis also remained guarded. Our study found that age-related macular degeneration was the most common underlying pathology, which is common as 12% of patients with this condition experience subretinal haemorrhages during the course of the disease.[25] This might lead to additional macular atrophy and scarring, as well as photoreceptor damage leading to reduced visual recovery. Therefore, patients could generally be informed that the intervention is intended to improve the condition, but due to scarring, most patients should expect a slight or no improvement in their visual acuity, particularly in age-related macular degeneration [3].

Though it is difficult to determine the superiority of any treatment option in most trials [22], complications after intervention are common [26]. When choosing a treatment option, it is important to consider the invasiveness and potential complications, particularly in the peripheral retina, which can affect daily activities [27, 28]. Our study found comparable visual outcomes and postinterventional haemorrhage size in all treatment groups regardless of the invasiveness, but with a high rate of complications and repeat surgery after the initial procedure. Out of the 45 patients being treated following the “Manchester protocol”, only 14 had to receive a secondary “rescue” vitrectomy, therefore sparing 69% in the treatment group the more invasive procedure while maintaining a stable visual result in comparison to the other groups. Group 5 however, treated by ppV and subretinal lavage, showed the largest expansion of subretinal blood and worst BCVA preinterventional, though the blood reduction through a highly invasive procedure did not lead to a statistically significant visual gain. Research should investigate whether a sequential approach as demonstrated in the “Manchester protocol” is more effective in terms of visual improvement and complication prevention compared to a more invasive initial procedure. Time is a crucial factor for treatment of subretinal hemorrhage [29]. Therefore, some authors may argue that delay of treatment by starting with a less invasive treatment at first, might affect the visual outcome. We could not observe this notion in our data, as patients treated within the “Manchester-Protocol” developed better final BCVA than patients undergoing primary vitrectomy.

As some studies suggest, anti-VEGF could also have theoretically favorable advantages compared to surgical approaches, especially in terms of efficacy, complications, and cost [6].

Although our study protocol demanded all patients to be treated, it should be noted that randomized trials and observational studies including untreated patients have also shown positive results [22]. In a randomised prospective submacular surgery trial, 454 patients received either removal of neovascularization and haemorrhage by pars plana vitrectomy or no treatment [26]. 44% of the operated eyes had improvement or stabilisation of visual acuity, which was not statistically significant. However, a significantly higher number of patients (5%) in the intervention group had retinal detachment compared to 1 patient in the observation group. Patients treated in the submacular surgery study mostly suffered from choroidal neovascularisation in age-related macular degeneration before the use of anti-VEGF. Of note, haemorrhages with other underlying diagnoses such as polypoidal choroidal vasculopathy [30] or retinal artery macroaneurysm [31] had a favorable outcome with observation only in case series. Future prospective studies are needed to confirm these findings and further evaluate the primary use of anti-VEGF as a less invasive treatment option.

It should be noted that this study has potential limitations due to its retrospective nature and the relatively small number of cases available for multi-group comparison. In addition, there were differences in group size between treatment modalities, with fewer cases in the more invasive treatment groups. Follow-up also varied, and surgery was performed by different posterior segment surgeons. The underlying disease was mostly neovascular age-related macular degeneration, but other causes were also included, which reduces comparability with other trials. In addition, the size of the haemorrhage, intravitreal haemorrhage and cataract surgery during follow-up in 20% of cases must be considered as potential confounders of the results observed in this study.

In summary, this retrospective study suggests that the choice of an interventional approach can significantly influence visual prognosis. While more invasive techniques may not demonstrate a clear advantage in postoperative outcomes, they appear to carry a higher risk of secondary complications. This leads us to maybe consider a stepwise approach to invasiveness, as proposed by the Manchester protocol, which aligns with our findings and may help to avoid unnecessary surgeries while still providing meaningful visual improvements. Further research is warranted to explore whether less invasive treatment protocols could potentially achieve similar functional outcomes with reduced complication rates.

## Supplementary Information

Supplementary Material 1. Supplementary Fig. 1: Comparative fundus photography and OCT images of the five treatment groups. Representative images from each group demonstrate the high intervariability in hemorrhage size and retinal thickness. Fundus photography highlights the surface appearance and distribution of hemorrhages, while OCT provides cross-sectional views of retinal layers, emphasizing the differences in thickness.

## Data Availability

All data generated or analyzed during this study are included in this article. Further enquiries can be directed to the corresponding author.
