# Supplementary figures and images for: Comparison of different treatment options in submacular haemorrhage
Source: BMC Ophthalmol. 2024 Dec 9;24:525. doi: 10.1186/s12886-024-03794-y (PMC11626755; doi:10.1186/s12886-024-03794-y)

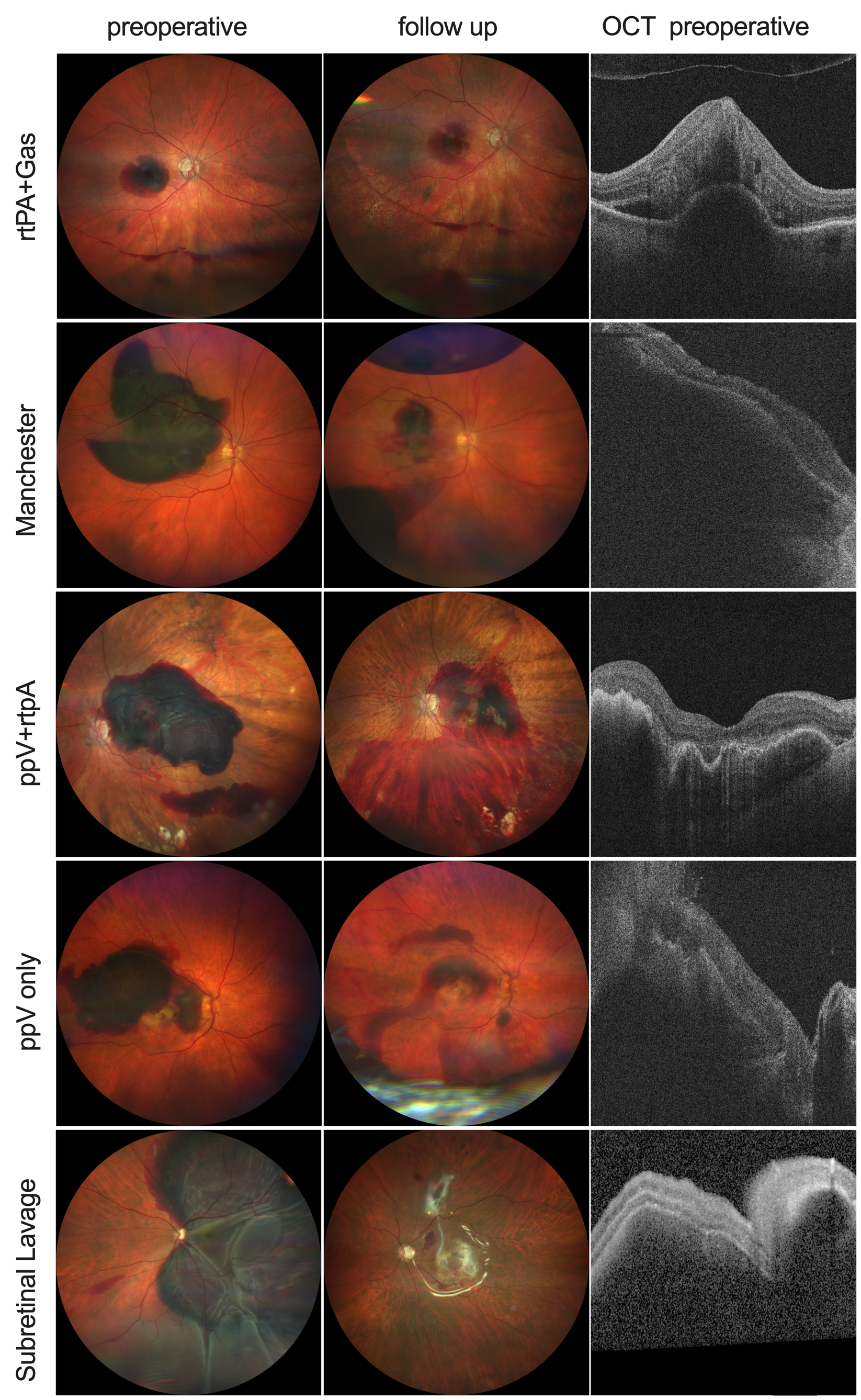

Supplement: Supplementary file 1 — Supplementary Material 1. Supplementary Fig. 1: Comparative fundus photography and OCT images of the five treatment groups. Representative images from each group demonstrate the high intervariability in hemorrhage size and retinal thickness. Fundus photography highlights the surface appearance and distribution of hemorrhages, while OCT provides cross-sectional views of retinal layers, emphasizing the differences in thickness. [file 12886_2024_3794_MOESM1_ESM.tiff]
